# Cocaine and Ischemic or Hemorrhagic Stroke: A Systematic Review and Meta-Analysis of Clinical Evidence

**DOI:** 10.3390/jcm12165207

**Published:** 2023-08-10

**Authors:** Luis F. Rendon, Stephanie Malta, Jacob Leung, Rafael Badenes, Ala Nozari, Federico Bilotta

**Affiliations:** 1 Department of Anesthesiology, Boston Medical Center, Boston, MA 02118, USA; luis.rendon@bmc.org (L.F.R.); malta@bu.edu (S.M.); jleung37@bu.edu (J.L.); ala.nozari@bmc.org (A.N.); 2 Department Anesthesiology and Surgical-Trauma Intensive Care, Hospital Clínico Universitario de Valencia, University of Valencia, 46010 Valencia, Spain; 3 Department of Anesthesiology and Critical Care, Sapienza University of Rome, 00185 Rome, Italy; bilotta@tiscali.it

**Keywords:** cocaine, ischemic stroke, hemorrhagic stroke, stroke, intracerebral hemorrhage, subarachnoid hemorrhage, mortality

## Abstract

Cocaine consumption has increased over the last decade. The potent sympathomimetic effects of the drug can lead to serious neurovascular complications in the form of ischemic stroke (IS), intracerebral hemorrhage (ICH), or subarachnoid hemorrhage (SAH). This systematic review and meta-analysis were designed to describe the clinical features and outcomes of patients suffering from IS, ICH, or SAH occurring in the context of cocaine use. The PubMed, Embase, Cochrane, and Web of Science libraries were queried in December 2022. Studies were included if they provided information regarding the epidemiology, clinical presentation, or outcomes in cocaine-associated strokes. Odds ratios (OR) were pooled using a random-effects model. A total of 36 papers were included. Strokes associated with cocaine use were more prevalent in younger populations and those of African American descent. Cocaine use increased the odds of IS, ICH, or SAH (OR = 5.05, *p* < 0.001). The odds of mortality (OR = 1.77, *p* = 0.0021), vasospasm (OR = 2.25, *p* = 0.0037), and seizures (OR = 1.61, *p* < 0.001) were also worse when strokes were associated with cocaine use. In addition to counseling patients on the benefits of drug cessation, clinicians should remain vigilant of the potential complications in patients who are hospitalized with cocaine-associated strokes.

## 1. Introduction

Cocaine is an addictive psychoactive stimulant and one of the most commonly abused drugs. According to the United Nations Office on Drug and Crime, an estimated 20 million adults worldwide used cocaine annually between 2010 and 2019 [1,2]. The pervasive use of cocaine is fueled by its addictive properties, which are primarily due to the inhibition of reuptake of dopamine in the nucleus accumbens [3,4]. However, cocaine also functions as a potent sympathomimetic substance by increasing the concentrations of serotonin and norepinephrine [4]. The resultant rise in catecholamines produces adrenergic effects that present as tachycardia and hypertension [3,5]. Other consequences of cocaine use include impaired platelet function, endothelial damage, and the release of pro-inflammatory mediators [6].

Cocaine can result in serious cardiovascular complications such as myocardial infarction and cardiac arrhythmias. Serious neurovascular complications have also been documented in the form of ischemic and hemorrhagic strokes, which includes intracerebral hemorrhage (ICH), intraventricular hemorrhage (IVH), and subarachnoid hemorrhage (SAH) [7]. In 2014, a systematic review was published suggesting that cocaine use increases the risk of both ischemic and hemorrhagic strokes [7].

The intricate pathophysiological pathways implicated in the onset of stroke following cocaine use incorporate an array of interconnected mechanisms. First, cocaine can cause transient elevation in blood pressure, as described above, and thus precipitate a hypertensive crisis, increasing the risk of cerebral hemorrhage. Second, the substance can induce vasospasm, leading to ischemia and the potential for resultant hemorrhages when the spasm is relieved. Third, cocaine use can lead to disruption of the vascular endothelium and alterations in blood coagulation pathways, making vessels more prone to rupture and bleeding. The hypertensive crisis and vasospasm induced by cocaine use not only increase the risk of hemorrhagic stroke but can also lead to ischemic stroke due to reduced blood flow to certain areas of the brain. Furthermore, the effect of cocaine on platelet aggregation could potentially contribute to the formation of thrombi, leading to ischemic events [6,8]. Hence, investigating both hemorrhagic and ischemic stroke types in this context is needed for a more comprehensive understanding of cocaine’s impact on cerebrovascular health. Despite this understanding, there is currently no systematic review comparing the clinical parameters and outcomes of cocaine-associated strokes to those due to long-standing cardiovascular disease.

Therefore, the aim of this systematic review is to examine the published literature to understand the clinical features of patients who suffer from cocaine-associated strokes and describe the outcomes after ischemic or hemorrhagic stroke in this population.

## 2. Materials and Methods

### 2.1. Search Strategy

The libraries of PubMed, EMBASE, and Web of Science were queried for relevant articles in accordance with the Preferred Reporting Items for Systematic Reviews and Meta-Analyses (PRISMA) guidelines [9]. This study was also registered in the International Prospective Register of Systematic Reviews (PROSPERO registration number CRD42022378254). The query was conducted in December 2022. A professional librarian was consulted to verify that the search syntax contained all synonyms for cocaine, ischemic stroke, and hemorrhagic stroke. The search syntax can be found in the Appendix A. Titles and abstracts of relevant studies were independently screened by two authors (S.M. and J.L.). Full-text articles were screened by two independent authors (S.M. and J.L.). Any discrepancies were resolved by a third author (L.F.R.). Prior reviews were also screened to identify additional papers that were missed by our search syntax.

### 2.2. Inclusion Criteria and Exclusion Criteria

Studies were considered for inclusion if they were published, peer-reviewed, and original articles. All study designs were eligible for inclusion in the study, but only studies that reported clinically relevant information or outcomes in patients presenting with ischemic or hemorrhagic stroke in the setting of cocaine use were selected. No restrictions were placed by date of publication. Criteria for exclusion were non-English publications, studies on subject <18 years of age, studies on non-human subjects, and studies with less than five participants. Articles that did not distinguish cocaine from other illicit substances were also excluded, as were previously published reviews.

### 2.3. Data Extraction

The following variables were collected from each of the included studies: first author name, year of publication, design and timing, type(s) of stroke evaluated, study period, number of patients, number of cocaine users, method of cocaine use detection, percent of male participants, and the mean or median age of the subjects. Variables related to the epidemiology and clinical presentation were qualitatively pooled for each study. When possible, the odds ratio (OR) and 95% confidence intervals (95% CIs) related to risk of stroke, risk of intraventricular hemorrhage, mortality, bad neurological outcome, vasospasm, ventriculostomy, tracheostomy, seizures, deep venous thrombosis, and percutaneous endoscopic gastrostomy tube were collected. Bad neurological outcome was defined as Modified Rankin Score (mRS) greater than three, Cerebral Performance Category (CPC) > 2, Glasgow Outcome Scale < 3, or as defined by the study authors. If ORs were not provided, we used the reported data to calculate the OR and their respective 95% CIs.

### 2.4. Data Analysis

Studies reporting similar outcomes were grouped together. Odds ratios for each outcome were weighed using a random-effects model with a DerSimonian-Laird (DL) estimator to obtain pooled effect sizes and the estimated 95% CIs. Forest plots were generated to summarize the results. Heterogeneity was assessed using the I^2^ statistic. Low heterogeneity was defined as I^2^ values between 0–25%, moderate heterogeneity between 25–50%, and substantial heterogeneity >50%. Data were analyzed in R (Version 4.2.2) [10].

### 2.5. Risk of Bias Assessment

Publication bias was assessed graphically using funnel plots and statistically using both the Begg rank correlation test and the Egger regression test. Only outcomes with ≥10 studies were examined for publication bias. Statistically significant publication bias was further explored with Duval and Tweedie’s trim and fill method. Risk of bias assessment was conducted using the Cochrane Risk of Bias in Non-Randomized Studies–of Interventions (ROBINS-I) [11].

## 3. Results

**Figure 1:**
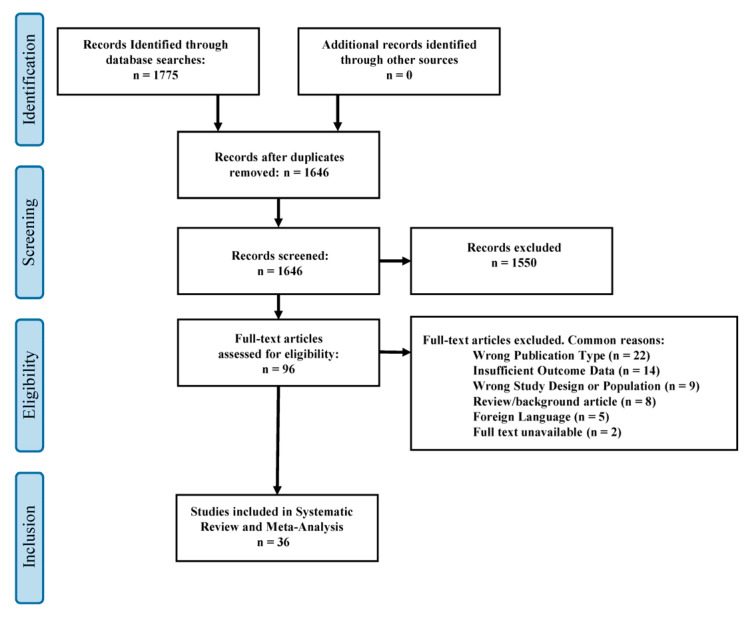
PRIMSA flow diagram depicting the study selection process.

The initial literature search identified 1775 records (Figure 1). After removing duplicates, 1646 potential articles were selected for screening. Upon review of study titles and abstracts, 96 of the above articles met the criteria for full-text assessment. From this pool, 36 eligible studies were included in this systematic review and meta-analysis (Table 1). The designs of the studies we included varied, consisting of 16 case-control studies, 11 retrospective cohorts, 4 prospective cohorts, 4 autopsies, and 1 comparative case series. The 36 selected articles collectively represent a patient population of 721,702 individuals, among whom 6281 were identified as cocaine users. Subarachnoid hemorrhage was the most common stroke type reported on, with 24 articles investigating this pathology. ICH followed closely, featuring in 18 studies, while ischemic stroke was explored in 14 articles, making it the third commonly studied stroke type.

### 3.1. Epidemiology

In the context of recent cocaine use, patients presenting with IS, ICH, or SAH were overall younger compared to those experiencing strokes due to other etiologies [16,20,28,29,32,33,34,42]. However, there were discrepancies in the literature. While two articles found no age difference at the time of stroke onset [8,42], one study suggested that an older age at stroke onset was associated with cocaine use [40].

Seven studies examined racial disparities, with six identifying a significant association between African American ethnicity and strokes related to cocaine use [8,28,29,33,36,40,41,42]. Gender differences were noted in four studies, three of which reported a higher incidences of strokes in males [28,30,36], while one found a higher incidence in females after recent cocaine use [40]. As for concurrent substance use, patients with cocaine-associated strokes were more likely to be tobacco smokers or use other illicit substances at the time of their presentation [20,29,30,32,35,39,42].

**Figure 2:**
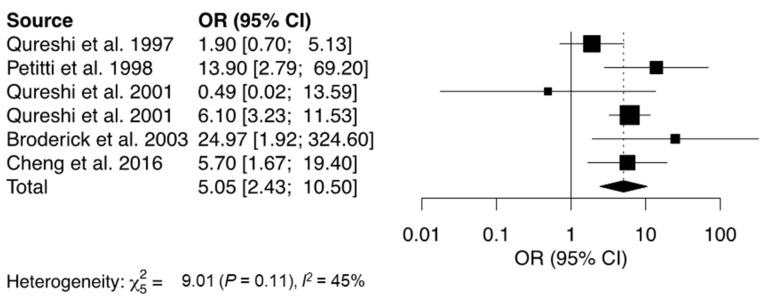
Forest plot showing the association between cocaine use and risk of stroke. The solid squares represent the point estimate of each study with the horizontal lines denoting the 95% CI. The size of the square is proportional to the relative weight of each respective study. The center of the diamond is the pooled estimate using a random-effects model and its width reflects the 95% confidence interval. OR = Odds Ratio, CI = Confidence Interval [17,18,21,22,23,39].

Recent cocaine use prior to stroke onset was significantly associated with an increased odds of IS, ICH, or SAH (OR = 5.12, Figure 2) [17,18,21,22,23,39]. One study focusing on patients with reversible cerebral vasoconstriction syndrome found that cocaine use was a significant risk factor, leading to a significantly higher incidence of ICH (OR = 3.11, 95%CI = 2.80–9.35), SAH (OR = 2.36, 95%CI = 1.05–5.30), and IPH (OR = 5.22, 95%CI = 1.70–15.97) [44].

### 3.2. Clinical Presentation

The risk of experiencing an ICH was found to be more prominently increased with recent cocaine use than the risk of an ischemic stroke. [30,31] A direct correlation was reported between the concentrations of cocaine or cocaine-related metabolites in the body and the incidence of ICH or SAH [43]. In contrast, stroke severity measures at the time of admission, including Hunt and Hess (HH) score, Fisher grade, World Federation of Neurological Surgeons (WFNS) grade, and the proportion of patients with a Glasgow Coma Score < 8, showed no statistically significant difference between cocaine users and non-users across five studies [20,26,29,34,35]. Yet, three studies reported more severe HH scores upon admission in patients enduring cocaine-associated SAH [12,24,27]. One study drew an association between a positive toxicology upon admission and stroke severity [43]. Interestingly, the severity of ischemic stroke, as gauged by the NIH Stroke Scale, was not distinctively different between cocaine users and non-users [16,33,36,41].

Measures of coagulopathy, namely PT or PTT, were not different between cocaine users and non-users [42]. Despite this, thromboelastography values suggested decreased clotting time and clot strength in cocaine-positive patients [29].

Imaging results from patients with aneurysmal SAH suggested a more frequent occurrence of aneurysms in the proximal sections of the anterior circulation in cocaine users, as compared to their location in the posterior circulation [19,20,27]. Further, the total number of aneurysms detected was higher in patients who used cocaine compared to non-users, whereas the average diameter of all aneurysms was smaller in cocaine users [16,19,26,27]. With respect to ICH location, one study reported a higher number of hemorrhages in subcortical locations, with the basal ganglia being the most common site [33]. Another study failed to find a relationship between ICH location and cocaine use [41]. An article on cocaine-associated IS and ICH found a higher number of strokes contained within the middle cerebral artery territory compared to other locations in the neurovasculature [30]. In cases of hemorrhagic strokes (SAH or ICH), the odds of intraventricular hemorrhage (IVH) was not significantly elevated in cocaine users (OR = 1.71) [8,34,41].

Pathological examinations of the cerebral vasculature in cocaine users who presented with SAH or ICH revealed an absence of disease states such as vasculitis or other specific cerebral pathologies [14,15]. Quantitative analysis of cerebral arterial diameter measurements also did not reveal a difference between cocaine-positive and cocaine-negative patients [25]. Despite this, one study reported that patients who used cocaine had more intracranial vascular abnormalities, as opposed to extracranial, detected by radiological scans [25]. Postmortem pathological examinations on patients who died from hemorrhagic strokes in the context of recent cocaine use revealed a higher incidence of hypertensive cerebrovascular disease if the patient had suffered an ICH, as compared to SAH from aneurysmal rupture [13].

### 3.3. Clinical Outcomes

**Figure 3:**
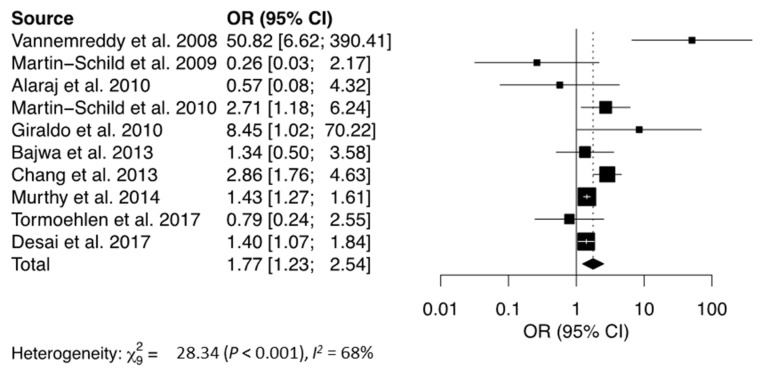
Forest plot showing the association between cocaine use and mortality following stroke. The solid squares represent the point estimate of each study with the horizontal lines denoting the 95% CI. The size of the square is proportional to the relative weight of each respective study. The center of the diamond is the pooled estimate using a random-effects model and its width reflects the 95% confidence interval. OR = Odds Ratio, CI = Confidence Interval [8,27,28,29,30,33,36,40,41].

**Figure 4:**
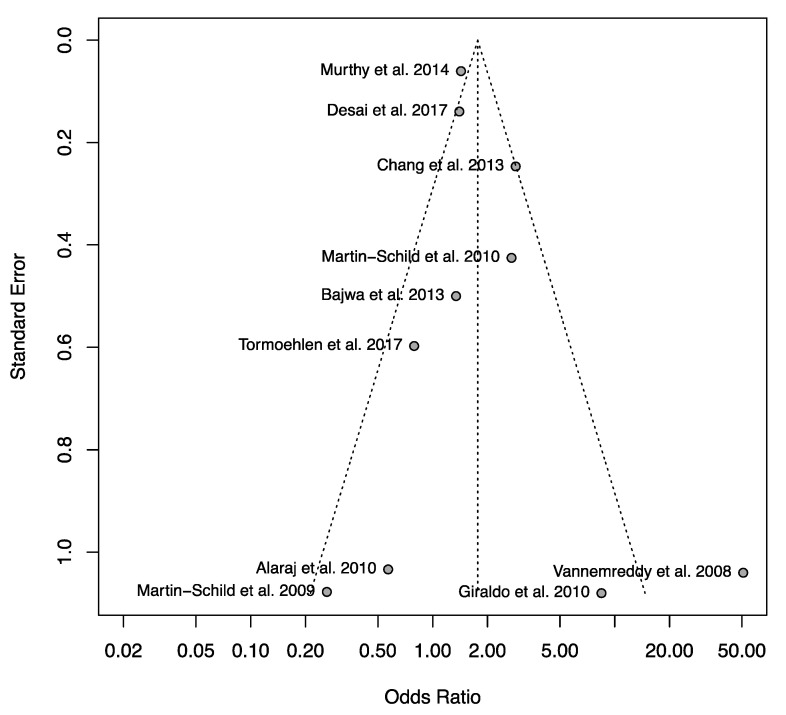
Funnel plot from the pooled studies reporting outcomes for mortality after stroke in the setting of cocaine use. There was no significant asymmetry found. Egger’s and Begg’s test were negative for publication bias [8,27,28,29,30,33,34,36,40,41].

**Figure 5:**
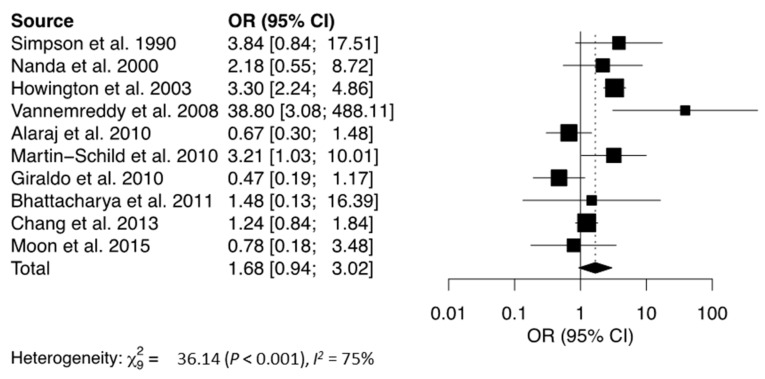
Forest plot showing the association between cocaine use and bad neurological outcome following stroke. The solid squares represent the point estimate of each study with the horizontal lines denoting the 95% CI. The size of the square is proportional to the relative weight of each respective study. The center of the diamond is the pooled estimate using a random-effects model and its width reflects the 95% confidence interval. OR = Odds Ratio, CI = Confidence Interval [8,12,19,24,27,29,30,32,34,37].

**Figure 6:**
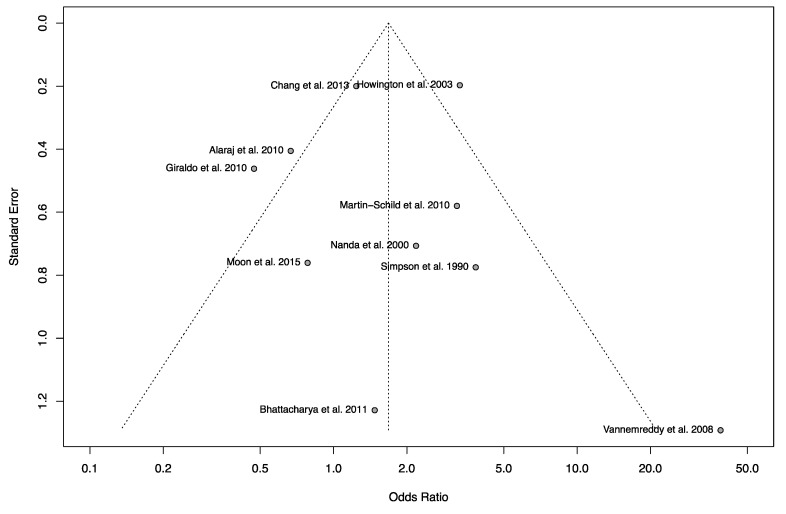
Funnel plot from the pooled studies reporting outcomes for mortality after stroke in the setting of cocaine use. There is slight asymmetry in the plot. Egger’s and Begg’s test were negative for publication bias [8,12,19,24,27,29,30,32,34,37].

The mortality rate observed following IS, ICH, or SAH was significantly higher in cocaine users when compared to non-users (OR = 5.12, Table 2, Figure 3) [8,27,28,29,30,33,34,36,40,41]. The funnel plot did not reveal any asymmetry (Figure 4). Egger’s and Begg’s test did not reveal any significant publication bias. There was no significant relationship between cocaine use and bad neurological outcome (OR = 1.68, Figure 5) [12,19,24,27,28,29,30,32,34,37]. The funnel plot was suggestive of slight asymmetry (Figure 6), but Egger’s and Begg’s test were negative for significant publication bias. One study further failed to find a difference in NIH Stroke Scale at the time of discharge between cocaine users and non-users [35]. Following stroke, cocaine users were at increased odds of having seizures compared to non-users (OR = 1.61) [32,36,38]. In cases of SAH, cocaine use was significantly associated with vasospasm (OR = 2.25) [20,24,29,36,46], with one study reporting a higher rate of aneurysm re-rupture in cocaine users (OR = 3.00) [34]. There was no difference in the rates of DVT between cocaine users and non-users (OR = 1.10) [32,36,40]. There was also no association between cocaine use and the need for ventriculostomy (OR = 1.04) [8,33,36], tracheostomy (OR = 0.88) [33,36,41], or need for percutaneous endoscopic gastrostomy tube (OR = 0.82) following stroke [36,41].

### 3.4. Risk of Bias Assessment

**Figure 7:**
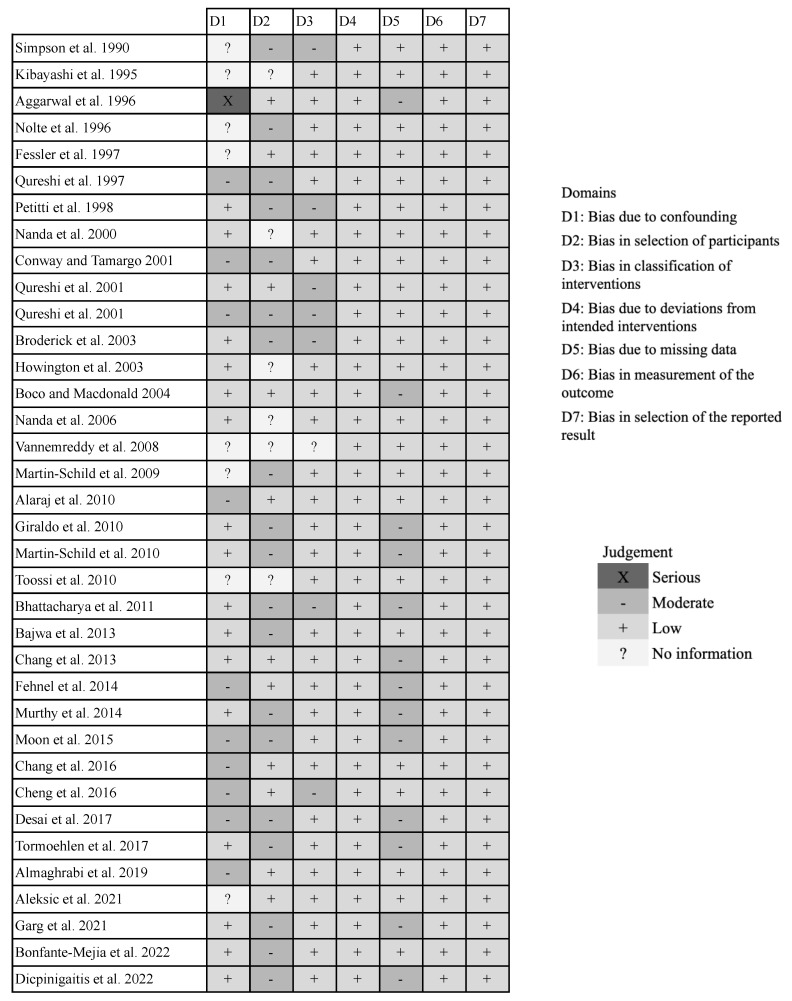
Results from the risk of bias assessment [8,12,13,14,15,16,17,18,19,20,21,22,23,24,25,26,27,28,29,30,31,32,33,34,35,36,37,38,40,41,42,43,44,45,46].

Bias due to confounding was serious in 3%, moderate in 27%, low in 46%, and unable to be determined in 22% of studies (Figure 7). The most common reason for confounding bias was a lack of control for other common stroke risk factors found in cocaine users, especially smoking and hypertension. Bias due to participant selection was moderate in 53%, low in 31%, and unable to be determined in 17% of studies. This form of bias was due to the retrospective nature of most studies. Cocaine users were identified as patients who either underwent urine drug screenings indicative of cocaine use or self-reported their consumption. However, not all stroke patients were subjected to such screenings or standardized interviews to confirm cocaine use. This limitation presents a moderate risk of excluding potentially eligible participants from the study. This potential for bias was further compounded by the possibility of missing records, where some patients’ data were not accessible. Such omissions could further contribute to the inadvertent exclusion of eligible participants from the study.

In terms of intervention classification bias, 19% of the studies exhibited a moderate level while 78% showed a low level, with an indeterminate level in 3% of the studies. This bias mainly stemmed from the reliance on self-reported cocaine use, rather than verified urine drug screening, potentially leading to misclassification of recent cocaine use. Bias resulting from deviations from the intended interventions was notably absent, being low across 100% of the studies. The intervention, which was cocaine use, remained consistent throughout all studies. Bias related to missing data was moderate in 36% and low in 64% of the studies. This bias primarily arose due to incomplete data regarding the history of cocaine use, such as dosage and timing specifics. Measurement bias concerning the outcome was negligible in all studies, indicating that all studies were consistent and precise in gauging clinical outcomes. Similarly, bias in the selection of the reported result was minimal across all studies, with each study reporting all of its respective outcomes.

## 4. Discussion

This systematic review and meta-analysis originally and comprehensively examine the literature and describe how strokes associated with cocaine use differ in epidemiology, clinical presentation, and outcomes from those due to other etiologies. The observed findings indicate that cocaine use is a potent risk factor for ischemic or hemorrhagic stroke, especially among African Americans or younger patients. In cases of SAH, cocaine use predisposed patients to developing smaller, albeit more numerous, aneurysms that were located in the proximal portion of the anterior circulation. The results further suggest that IS, ICH, or SAH occurring in the setting of cocaine use are associated with an increased odds of mortality, vasospasm, and seizures.

The finding that cocaine use is associated with mortality in patients who suffer from stroke has important implications. Cocaine use is known to promote arterial vasoconstriction, thrombus formation, hypertension, and increased vascular shearing forces. Through those mechanisms, acute cocaine use has similarly been associated with increased mortality from serious cardiovascular events, including myocardial infarction and aortic dissection [47,48,49]. Unfortunately, the data presented by the studies included in this systematic review and meta-analysis were insufficient to differentiate between neurological or cardiovascular causes of death. Cocaine users who suffered strokes were at increased odds of developing seizures and vasospasm, two complications that are associated with secondary, and potentially preventable, brain injury and death in the post-stroke period. Therefore, cocaine users who suffer IS, ICH, or SAH may benefit from additional monitoring, including continuous electroencephalography (EEG) monitoring, and routine transcranial doppler in cases of SAH. Clinicians may also consider anti-epileptic medication in critically ill patients suffering from cocaine-induced strokes. Additionally, given the constrained evidence supporting the use of transcranial doppler for detecting vasospasm in arteries beyond the middle cerebral artery, it becomes important to strive for optimal cerebral perfusion pressures in patients with subarachnoid hemorrhage. This proactive measure can help in mitigating the effects of delayed cerebral ischemia [50]. If vasospasm is suspected, expeditious angiography should be conducted with the administration of intravenous or intra-arterial vasodilators, or performance of angioplasty, when indicated.

In the setting of SAH, cocaine users presented with more numerous aneurysms that were of smaller diameter compared to non-users. Hemodynamic fluctuations from repeated cocaine consumption may therefore promote early aneurysm formation. The presence of multiple aneurysms presents a challenging issue for clinicians to address [51]. A recent meta-analysis found that patients with multiple intracranial aneurysms are at increased risk for the growth and rupture of existing, as well as the development of new, aneurysms [51,52]. Despite this, aneurysms located in the anterior circulation, as was the predominant cite in our study, may be protective against rupture [53]. However, acute changes in cerebral hemodynamics, especially from acute cocaine intoxication, that may precipitate aneurysm rupture are difficult to account for. Ultimately, cocaine users found to have multiple intracranial aneurysms should be counseled on cessation of drug consumption as well as repeat imaging to monitor for aneurysm growth and rupture, with treatment of aneurysms that present with high-risk features, such as irregular shape, size > 5 mm, and an aspect ratio >1.3 [52,54,55].

Determination of cocaine use varied across studies. There was a predominant reliance on either self-reported use and/or positive toxicology. The lack of standardized screening methods likely contributed to heterogeneity in our meta-analyses and bias, as relying on self-reported substance use patterns introduces recall bias. Studies in the future should focus on screening patients by positive toxicological examinations when selecting their cohorts. Importantly, a large number of papers reported co-existing substance use disorders, especially tobacco and alcohol, or toxicology results consistent with polypharmacy in patients presenting with cocaine-associated strokes. Recent studies have estimated that 70–80% of cocaine users also abuse other substances, supporting the need for substance use screening and counseling for these patients during their hospitalization [56,57].

The biggest limitation to our study is the quality of evidence, which made it impractical to stratify our analyses by stroke type. There were few papers reporting outcomes by either IS, ICH, or SAH alone, with many studies including some combination of these. Further, a large portion of studies did not control for important covariates that could influence the risk of stroke or mortality and morbidity associated with it. Nonetheless, our inclusion of a wide variety of study designs allowed us to explore important clinical characteristics that may have otherwise been overlooked.

The search syntax we employed encompassed all synonymous terms for ischemic and hemorrhagic strokes but did not include general cardiovascular outcomes. As such, studies reporting the incidence of ischemic or hemorrhagic strokes as a part of a broader cardiovascular disease spectrum were not incorporated into our meta-analysis.

A subsequent, separate search highlighted a recent study exploring the long-term risk of cardiovascular disease following cocaine use in women [58]. The study’s authors suggested that cocaine use might be linked to an enduring risk of cerebrovascular disease, characterized by both ischemic and hemorrhagic strokes, which can occur even up to 15 years after cocaine use.

## 5. Conclusions

Cocaine consumption and associated mortality have risen over recent years. As such, it is increasingly important for clinicians to recognize how neurovascular diseases present in this patient population. This systematic review and meta-analysis found that cocaine use is associated with a higher risk of IS, ICH, or SAH. Strokes that occur in the context of cocaine use are also associated with increased mortality, seizures, and vasospasm. Future studies should address risk factors and dedicated diagnostic and procedural workup in patients presenting with IS, ICH, or SAH associated with recent cocaine consumption. It is vital to communicate accurate and clear information to healthcare professionals and the general public, underscoring the significant health risks associated with cocaine consumption, particularly as they relate to intracranial vascular disease.

## Figures and Tables

**Table 1:** Characteristics of included studies.

First Author and Year	Design and Timing	Strokes Evaluated	Study Period	Number of Patients	Number of Patients with Cocaine Use	Cocaine Use Detection Method	% Male	Age (Mean, Years)
Simpson et al., 1990 [12]	Retrospective Cohort	SAH	1980–1987	150	17	Witnessed	Cocaine positive: 53% Cocaine negative: --	Cocaine positive: 34 Cocaine negative: --
Kibayashi et al., 1994 [13]	Autopsy	ICH, SAH	1991–1994	52	52	Positive toxicology of blood, bile, or urine	65%	ICH: 41 aSAH: 39
Aggarwal et al., 1996 [14]	Autopsy	ICH, SAH	1986–1990	14	14	Positive toxicology or history of cocaine use or abuse	50%	40
Nolte et al., 1996 [15]	Autopsy	SAH, ICH	1989–1990	17	10	Positive toxicology of specimens	Cocaine positive: 80% Cocaine negative: 29%	Cocaine positive: 39 Cocaine negative: 43
Fessler et al., 1997 [16]	Retrospective Cohort	IS, ICH, SAH	1988–1993	33	33	Positive toxicology and confirmation by history	52%	37
Qureshi et al., 1997 [17]	Case Control	IS, ICH, SAH	1990–1994	291	54	Positive toxicology or self-reported on admission	Stroke group: 58% Control group: 56%	Stroke group: 33 Control group: 33
Petitti et al., 1998 [18]	Case Control	IS, ICH, SAH	1991–1994	1368	27	Self-reported within 7 days	0%	Cocaine positive: 33 Cocaine negative: 37
Nanda et al., 2000 [19]	Retrospective cohort	aSAH	--	149	14	Self-reported	Cocaine positive: 36% Cocaine negative: 64%	Cocaine positive: 38 Cocaine negative: 49
Conway and Tamargo 2001 [20]	Case Control	aSAH	1992–1999	440	27	Positive toxicology on admission	Cocaine positive: 18% Cocaine negative: 29%	Cocaine positive: 36 Cocaine negative: 52
Qureshi et al., 2001 [21]	Case Control	ICH	1990–1997	488	101	Self-reported	61%	39.2
Qureshi et al., 2001 [22]	Case Control	IS, ICH, SAH	1988–1994	10,085	Infrequent users: 731 Frequent users: 532	--	Infrequent users: 59% Frequent users: 67% Cocaine negative: 44%	Infrequent users: 31 Frequent users: 33 Cocaine negative: 33
Broderick et al., 2003 [23]	Case control	aSAH	1994–1999	702	9	--	Cases: 39% Controls: 39%	--
Howington et al., 2003 [24]	Retrospective cohort	aSAH	1996–2001	108	36	Positive toxicology on admission	Cocaine positive: 67% Cocaine negative: 25%	Cocaine positive: 34 Cocaine negative: 40
Boco and Macdonald 2004 [25]	Prospective Cohort	SAH	1993–2003	39	13	Positive toxicology on admission	--	--
Nanda et al., 2006 [26]	Case series	SAH, IS, ICH	--	42	42	ICD codes	60%	38
Vannemreddy et al., 2008 [27]	Case control	aSAH	--	64	129	Self-reported	Cocaine positive: 38% Cocaine negative: 38%	Cocaine positive: 32 Cocaine negative: 50
Martin-Schild et al., 2009 [28]	Retrospective cohort	IS	2004–2007	87	29	Positive toxicology	Cocaine positive: 83% Cocaine negative: 61%	Cocaine positive: 48 * Cocaine negative: 55 *
Alaraj et al., 2010 [29]	Retrospective Cohort	SAH	2002–2007	600	31	Positive toxicology or self-reported within 72 h	Cocaine positive: 39% Cocaine negative: 61%	Cocaine positive: 45 Cocaine negative: 54
Giraldo et al., 2010 [30]	Case Control	IS, ICH	2004–2006	186	93	Positive toxicology or self-reported	Cases: 66% Controls: 41%	Cases: 49 Controls: 55
Martin-Schild et al., 2010 [8]	Retrospective Cohort	ICH	2004–2007	150	45	Positive toxicology at the time of admission	Cocaine positive: 60% Cocaine negative: 68%	Cocaine positive: 50 * Cocaine negative: 51 *
Toossi et al., 2010 [31]	Retrospective Cohort	IS, ICH, SAH	1998–2008	5142	96	Positive toxicology or self-reported within 14 days	Current users: 51% Prior users: 90%	Current users: 61 Prior users: 35
Bhattacharya et al., 2011 [32]	Retrospective Cohort	IS	2005–2007	262	41	Positive toxicology on admission	Cocaine positive: 65% Cocaine negative: 60%	Cocaine positive: 52 Cocaine negative: 59
Bajwa et al., 2013 [33]	Retrospective Cohort	ICH	2007–2009	102	20	Positive toxicology within 48 h of admission	Cocaine positive: 80% Cocaine negative: 67%	Cocaine positive: 51 * Cocaine negative: 57 *
Chang et al., 2013 [34]	Retrospective Cohort	SAH	1991–2009	1134	142	Positive toxicology or self-reported within 72 h	--	Cocaine positive: 49 Cocaine negative: 53
Fehnel et al., 2014 [35]	Case Control	IS	2007–2010	273	91	Positive toxicology or self-reported within 14 days	Cocaine positive: 74% Cocaine negative: 74%	Cocaine positive: 50 Cocaine negative: 50
Murthy et al., 2014 [36]	Case Control	aSAH	2007–2010	103,876	1702	ICD codes	Cocaine positive: 53% Cocaine negative: 39%	Cocaine positive: 46 Cocaine negative: 58
Moon et al., 2015 [37]	Prospective cohort	aSAH	2003–2007	398	37	Positive toxicology or self-reported	Methamphetamine positive: 29% Methamphetamine negative: 42%	Methamphetamine positive: 43 Methamphetamine negative: 55
Chang et al., 2016 [38]	Prospective cohort	aSAH	1991–2009	1134	142	Positive toxicology or self-reported within 72 h	28%	Seizures: 50 No seizures: 53
Cheng et al., 2016 [39]	Case Control	IS	1992–2008	2244	602	Self-reported	Cases: 54% Controls: 47%	Cases: 41 Controls: 39
Desai et al., 2017 [40]	Case Control	IS	--	584,115	1135	ICD codes	Cases: 48% Controls: 49%	Cases: 73 Controls: 70
Tormoehlen et al., 2017 [41]	Case Control	ICH	2009–2011	142	21	Positive toxicology	Cocaine positive: 62% Cocaine negative: 60%	--
Almaghrabi et al., 2019 [42]	Prospective Cohort	IS, ICH	2009–2014	91	8	Positive toxicology on admission	Cocaine positive: 63% Cocaine negative: 65%	Cocaine positive: 54 Cocaine negative: 58
Aleksic et al., 2021 [43]	Autopsy	ICH, SAH	2005–2018	26	26	Positive cocaine or metabolite in tissue samples	76%	34
Garg et al., 2021 [44]	Case Control	ICH, SAH	2016–2017	1834	118	--	24%	48
Bonfante-Mejia et al., 2022 [45]	Case Control	IS	2005–2015	174	37	Positive toxicology or self-reported	--	Cases: 55 Controls: 55
Dicpinigaitis et al., 2022 [46]	Case Control	ICH, SAH	2015–2018	5880	95	--	Vasospasm: 61% No Vasospasm: 49%	Vasospasm: 52 No Vasospasm: 56

SAH = Subarachnoid hemorrhage, ICH = Intracerebral hemorrhage, IS = Ischemic Stroke, aSAH = Aneurysmal subarachnoid hemorrhage, ICD = International Classification of Disease–Not available, * Median.

**Table 2:** Results of Meta-Analyses.

Outcome	OR	95% CI	I^2^	*p*-Value	Number of Studies [Citations]
Risk of Stroke	5.12	2.80–9.35	45%	<0.001	6 [17,18,21,22,23,39]
Risk of IVH	1.71	0.81–3.59	73%	0.16	3 [8,34,41]
Mortality	1.77	1.23–2.54	68%	0.0021	10 [8,27,28,29,30,33,34,36,40,41]
Bad neurological outcome	1.68	0.94–3.02	75%	0.081	10 [8,12,19,24,27,29,30,32,34,37]
Vasospasm	2.25	1.30–3.88	83%	0.0037	5 [20,24,29,36,46]
Ventriculostomy	1.04	0.44–2.45	76%	0.93	3 [8,33,36]
Tracheostomy	0.88	0.73–1.05	0%	0.15	3 [33,36,41]
Seizures	1.61	1.35–1.92	9%	<0.001	3 [32,36,38]
DVT	1.10	0.66–1.83	75%	0.71	3 [32,36,40]
PEG tube	0.82	0.65–1.04	0	0.099	2 [36,41]

OR = Odds Ratio, CI = Confidence Interval, IVH = Intraventricular Hemorrhage, DVT = Deep Venous Thrombosis, PEG = Percutaneous Endoscopic Gastrostomy.

## Data Availability

Data sharing not applicable.

## Appendix A

*Search Strings*

Pubmed

((Cocaine[Mesh] OR “Cocaine-Related Disorders”[Mesh] OR “Cocaine Smoking”[Mesh] OR Cocain* OR crack) AND (“Stroke”[mesh] OR “Subarachnoid Hemorrhage”[Mesh] OR “cerebrovascular accident*” OR “intracerebral hemorrhage*” OR “hemorrhagic stroke*” OR “haemorrhagic stroke*” OR “ischemic stroke*” OR “ischaemic stroke*” OR “subarachnoid hemorrhage*” OR “subarachnoid haemorrhage*” OR “embolic stroke*” OR “thrombotic stroke*” OR “lacunar stroke*” OR “Cryptogenic stroke*”))

Cochrane:

#1. (cocaine* OR crack): ti,ab,kw
#2. MeSH descriptor: [Stroke] explode all trees
#3. MeSH descriptor: [Subarachnoid Hemorrhage] explode all trees
#4. (“cerebrovascular accident*” OR “intracerebral hemorrhage*” OR “hemorrhagic stroke*” OR “haemorrhagic stroke*” OR “ischemic stroke*” OR “ischaemic stroke*” OR “subarachnoid hemorrhage*” OR “subarachnoid haemorrhage*” OR “embolic stroke*” OR “thrombotic stroke*” OR “lacunar stroke*” OR “Cryptogenic stroke*”): ti,ab,kw
#5. #2 OR #3 OR #4
#6. #1 AND #5

Embase

(‘cocaine’/exp OR ‘2beta carbomethoxy 3beta benzoxytropane’:ti,ab,kw OR ‘benzoylmethyl ecgonine’:ti,ab,kw OR ‘beta cocaine’:ti,ab,kw OR ‘cocain’:ti,ab,kw OR ‘cocain hydrochloride’:ti,ab,kw OR ‘cocaine’:ti,ab,kw OR ‘cocaine hydrochloride’:ti,ab,kw OR ‘cocaine methiodide’:ti,ab,kw OR ‘codrenine’:ti,ab,kw OR ‘crack (drug)’:ti,ab,kw OR ‘crack cocaine’:ti,ab,kw OR ‘ecgonine methyl ester benzoate’:ti,ab,kw OR ‘erythroxylin’:ti,ab,kw OR ‘goprelto’:ti,ab,kw OR ‘l cocaine’:ti,ab,kw OR ‘l ecgonine 3 benzoate 2 methyl ester’:ti,ab,kw OR ‘levo cocaine’:ti,ab,kw OR ‘locosthetic’:ti,ab,kw OR ‘methyl 3 benzoyloxy 8 methyl 8 azabicyclo [3.2.1] octane 2 carboxylate’:ti,ab,kw OR ‘neurocaine’:ti,ab,kw OR ‘numbrino’:ti,ab,kw OR ‘sterilocaine’:ti,ab,kw OR ‘cocaine dependence’/exp OR ‘addiction, cocaine’:ti,ab,kw OR ‘cocaine addiction’:ti,ab,kw OR ‘cocaine dependence’:ti,ab,kw OR ‘cocaine dependency’:ti,ab,kw OR ‘cocaine-related disorders’:ti,ab,kw OR ‘dependence, cocaine’:ti,ab,kw OR ‘cocaine smoking’/exp OR ‘cocaine smoking’:ti,ab,kw OR ‘crack smoking’:ti,ab,kw) AND (((h?emorrhagic OR isch?emic OR embolic OR thrombotic OR lacunar OR cryptogenic) NEAR/3 stroke*) OR ((intracerebral OR subarachnoid) NEAR/3 h?emorrhage*) OR ‘cerebrovascular accident*’)

Web of Science

(TS = (“Cocaine-Related Disorders” OR “Cocaine Smoking” OR Cocain* OR crack)) AND TS = (“Stroke*” OR “cerebrovascular accident*” OR “intracerebral hemorrhage*” OR “hemorrhagic stroke*” OR “haemorrhagic stroke*” OR “ischemic stroke*” OR “ischaemic stroke*” OR “subarachnoid hemorrhage*” OR “subarachnoid haemorrhage*” OR “embolic stroke*” OR “thrombotic stroke*” OR “lacunar stroke*” OR “Cryptogenic stroke*”)
